# CAR‐T‐OPENIA: Chimeric antigen receptor T‐cell therapy‐associated cytopenias

**DOI:** 10.1002/jha2.350

**Published:** 2021-11-21

**Authors:** Alankrita Taneja, Tania Jain

**Affiliations:** ^1^ Department of Medicine Roswell Park Comprehensive Cancer Center Buffalo New York USA; ^2^ Department of Oncology, Sidney Kimmel Comprehensive Cancer Center Johns Hopkins University Baltimore Maryland USA

**Keywords:** anemia, chimeric antigen receptor, neutropenia, thrombocytopenia

## Abstract

Chimeric antigen receptor (CAR) T‐cell is the most recent version in the evolution of cellular therapy with promising responses, which has revolutionized the management of some hematological malignancies in the current times. As the clinical use has progressed rather rapidly since the first approval in 2017, toxicities beyond cytokine release syndrome and immune effector cell‐associated neurological syndrome have surfaced. Cytopenias are common in <30 days (“early”), 30–90 days (“short‐term”) as well as >90 days (“prolonged”); and have clinical implications to patient care as well as resource utilization. We review the details of etiology, factors associated with cytopenias, and management considerations for patients with cytopenias for each of these time‐frames. This would potentially serve as a clinical guide for hematological toxicity or CAR‐T‐OPENIA, which is commonly encountered with the use of CAR T‐cell therapy.

**Abbreviations**
ALL: acute lymphoblastic leukemia
BCMA: B‐cell maturation antigen
BMT: bone marrow transplantation
CAR: chimeric antigen receptor
CRS: cytokine release syndrome
HLH: hemophagocytic lymphohistiocytosis
ICANS: immune effector cell‐associated neurotoxicity syndrome
IL: interleukin
SDF: stromal cell‐derived factor
TPO: thrombopoietin

## 1 INTRODUCTION

Chimeric antigen receptor (CAR) T‐cell therapy is a major breakthrough and has revolutionized treatment of B‐cell malignancies, while quickly establishing its role in other hematological and solid organ malignancies [1, 2, 3, 4, 5, 6, 7, 8, 9, 10]. Despite the initial encouraging responses, more common use has brought to surface several on‐target/off‐tumor and off‐target toxicities which remain imperative to understand for safe delivery of this therapy. Cytokine release syndrome (CRS), immune effector cell‐associated neurotoxicity syndrome (ICANS) and hypogammaglobulinemia have been reported in the above mentioned pivotal trials and explored extensively otherwise [11, 12, 13, 14, 15, 16, 17, 18, 19]. Subsequently, cardiopulmonary toxicity has been reported while at the same time, safety from a stand‐point of renal dysfunction as well as in the post‐allogeneic blood or marrow transplantation (BMT) setting has been described [20, 21, 22, 23].

More recently, delayed hematopoietic recovery has drawn increasing recognition in clinical practice and implications in the form of infections, resource utilization for transfusions, and limited options for salvage therapy [24, 25]. Hence, appropriate evaluation and management thereof is paramount to ensure safe and effective post‐CAR T‐cell infusion care of these patients. In this review, we discuss the various nuances for consideration when evaluating patients with cytopenias following CAR T‐cell therapy or CAR‐T‐OPENIA.

## 2 CAR‐T‐OPENIA IS REAL

### 2.1 What the pivotal trials and data, thus far, taught us?

With tisagenlecleucel for relapsed/refractory B‐cell acute lymphoblastic leukemia (ALL) in children and young adults, 41% patients had grade 3–4 thrombocytopenia, and 53% had grade 3–4 neutropenia that had not resolved by 30 days following CAR T‐cell infusion [8]. With brexucabtagene autoleucel for B‐cell ALL in adults, grade 3–4 thrombocytopenia was seen in 30% and neutropenia in 27% patients [3]. With the same product in mantle cell lymphoma, 94% patients had grade 3 or higher cytopenias making these the most common adverse events of this grade in ZUMA‐2 [2]. Similarly, in the three CD19 CAR T‐cell trials in relapsed/refractory B‐cell lymphomas, grade ≥3 neutropenia and thrombocytopenia lasting ≥28 days were frequently reported as shown in Table 1 [1, 4, 10]. CAR‐T‐OPENIA is not limited to studies in B‐cell lymphoma but were commonly reported, of grade 3–4, in the two B‐cell maturation antigen (BCMA) directed CAR T‐cell trials for relapsed/refractory multiple myeloma as detailed in Table 1 [6, 7]. The patterns and rates of CAR‐T‐OPENIA from each of these studies are summarized in Table 1 and suggest that this is not a disease‐ or target‐specific toxicity but a class effect from CAR T‐cell therapy.

**TABLE 1:** Summary of CAR‐T‐OPENIA as reported in pivotal trials

Clinical trial (CAR target, disease)	Anemia	Thrombocytopenia	Neutropenia	Leukopenia	Additional comments
ELIANA (CD19 CAR T‐cell, pediatric/young adult ALL) [8]	Grade 3–4: 4%	Grade 3–4: 7%	Grade 3–4: 11%	Grade 3–4: 9%	71% patients with grade 3–4 thrombocytopenia and 80% with grade 3–4 neutropenia had improved to grade 2 or lower at the time of last assessment.
ZUMA‐1 (CD19 CAR T‐cell, DLBCL) [1]	Grade 3–4: 43%	Grade 3–4: 38%	Grade 3–4: 78%	Grade 3–4: 29%	At 3 months, 17% of the patients had grade 3 or higher cytopenias including anemia (3%), thrombocytopenia (7%), and neutropenia (11%).
JULIET (CD19 CAR T‐cell, DLBCL) [4]	Grade 3–4: 39%	Grade 3–4: 28%	Grade 3–4: 33%	Grade 3–4: 31%	At day 28, 41% patients had unresolved grade 3–4 thrombocytopenia, and 24% had unresolved grade 3–4 neutropenia. At 3 months, 38% had unresolved grade 3–4 thrombocytopenia, and no patients had unresolved grade 3–4 neutropenia.
TRANSCEND (CD19 CAR T‐cell, DLBCL) [10]	Grade 3–4: 37%	Grade 3–4: 27%	Grade 3–4: 60%	Grade 3–4: 14%	At day 29, 37% of the patients had grade 3 or higher cytopenia. By day 90, recovery to grade 2 or lower seen in 82% (anemia), 62% (thrombocytopenia), and 84% (neutropenia).
ZUMA‐2 (CD19 CAR T‐cell, MCL) [2]	Grade 3–4: 50%	Grade 3–4: 51%	Grade 3–4: 85%	N.R	At day 90, 12% had persistent anemia while 16% patients had persistent thrombocytopenia and neutropenia.
ZUMA‐3 (CD19 CAR T‐cell, adult ALL) [3]	Grade 3–4:49%	Grade 3–4: 30%	Grade 3–4: 27%	Grade 3–4: 23%	At day 30, 36% of patients had grade 3 or higher cytopenia: anemia 7%, thrombocytopenia 18%, neutropenia 25%.
KarMMa (BCMA CAR T‐cell, Multiple Myeloma) [6]	Grade 3–4: 60%	Grade 3–4: 52%	Grade 3–4: 89%	Grade 3–4: 39%	Among patients with >1 month grade 3–4 cytopenia, recovery to grade 2 or lower occurred at a median: 2.1 months (range, 1.2‐13.8) for thrombocytopenia and 1.9 months (range, 1.2–5.6) for neutropenia.
CARTITUDE‐1 (BCMA CAR T‐cell, Multiple Myeloma) [7]	Grade 3–4: 68%	Grade 3–4: 60%	Grade 3–4: 95%	Grade 3–4: 61%	Patients with grade 3–4 cytopenias recovered to grade 2 or lower by day 30 in 59% (thrombocytopenia), and 70% (neutropenia).
Jain et al. Blood Advances 2020 [26]	Grade 3–4: 77%	Grade 3–4: 65%	Grade 3–4: 95%	Grade 3–4: 100%	Normalization at 1 month in 7% (hemoglobin), 23% (platelets), 30% (neutrophils), 13% (WBC count).

Abbreviations: ALL, acute lymphoblastic leukemia; BCMA, B cell maturation antigen; CAR, chimeric antigen receptor; DLBCL, diffuse large B cell lymphoma; MCL, mantle cell lymphoma; NR, not reported.

### 2.2 Data from the “real‐world”

In line with the data from pivotal trials, data from retrospective studies using various products also suggest a high incidence of cytopenias following CAR T‐cell infusion. In one of the larger studies from Memorial Sloan Kettering Cancer Center, details of severity, duration and associated factors were studied for 83 patients including those who received FDA‐approved (axicabtagene ciloleucel and tisagenlecleucel) and institutionally developed products (19‐28z CD19 CAR T‐cell for relapsed/refractory ALL and BCMA directed for relapsed/refractory multiple myeloma) [26]. Nadir for hemoglobin was 7.1 g/dl, platelets were 29.5 × 10^3^, absolute neutrophil count was 0 and white blood cell (WBC) count was 0.2 × 10^3^/μl. This nadir was noted rather quickly, commonly within the first week after CAR T‐cell infusion. In this study, “normalization” of hemoglobin was seen in 39% patients, platelets in 34%, neutrophil count in 71% and total WBC count in 39% patients at 3 months, while at 1 year, these respective proportions were 67%, 78%, 89% and 89%. “Recovery” of counts at 1 month was statistically associated with baseline cytopenias, CAR construct (count recovery more common with tisagenlecleucel), grade ≥3 CRS/ICANS, peak C‐reactive protein and peak ferritin on univariate analysis. Association of count recovery at 1 month was significant for grade ≥3 CRS/ICANS after adjustment for baseline cytopenia and CAR construct. Interestingly, only CAR construct was associated with absence of count recovery at 3 months. This study overall suggests a possible contribution of the inflammatory milieu toward early hematopoietic recovery, or lack thereof. While serial cytokine levels were studied in a limited sample of 43 patients, this was not significantly associated with count recovery.

Another important retrospective study elucidating pattern of cytopenias was done in patients who received locally manufactured CD19 CAR T‐cells with a CD28 co‐stimulatory domain with fludarabine and cyclophosphamide lymphodepletion in relapsed/refractory B‐cell malignancies [27]. In addition to the common and early occurrence of cytopenias, this study also demonstrated a “biphasic” pattern of cytopenias: the first phase was early in the first week, and the second phase occurred after 21 days, well beyond the direct impact from lymphodepletion chemotherapy. Prior allogeneic BMT and higher‐grade CRS correlated with late cytopenias. In this study, stromal derived factor (SDF)‐1 correlated with late neutropenia. SDF‐1 is a chemokine produced by stromal cells upon binding to its receptor on hematopoietic cells (CXCR4) retains neutrophils in the marrow leading to a drop in neutrophil count in the peripheral blood [28, 29]. Similar perturbations in SDF‐1 are noted with delayed neutropenia following rituximab therapy [28].

## 3 POSSIBLE ETIOLOGIES, EVALUATION, AND MANAGEMENT

**FIGURE 1:**
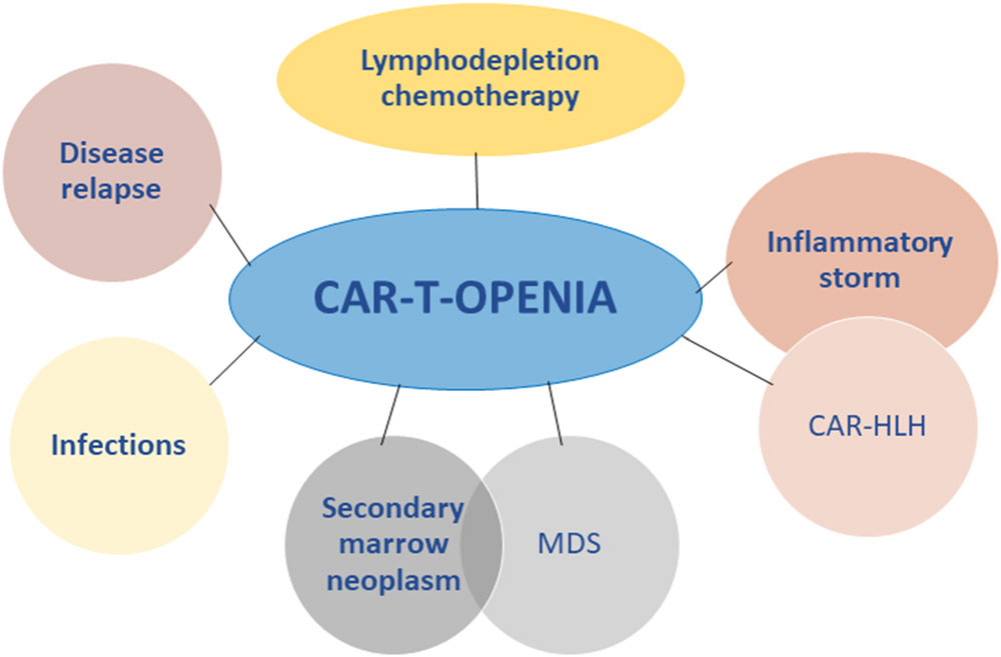
Possible etiology of Chimeric antigen receptor (CAR)‐T‐OPENIA

Figure 1 depicts the overall plausible etiologies of CAR‐T‐OPENIA to consider. For the purpose of this writing, we categorized the timeframe for hematopoietic toxicity from the day of CAR T‐cell infusion as early (<30 days), short‐term (30–90 days), and prolonged (>90 days). Possible etiologies, evaluation, and management considerations for each of these time‐frames are summarized in Table 2.

**TABLE 2:** Evaluation and management of CAR‐T‐OPENIA at various time‐frames following CAR T‐cell

	Early (<30 days)	Short‐term (30–90 days)	Prolonged (>90 days)
Possible etiologies	Lymphodepletion chemotherapy [34]	Lymphodepletion chemotherapy [34]	Disease relapse
	CRS/ICANS [17, 26, 27]	CAR‐HLH [34, 36, 37]	MDS [26, 49]
	CAR‐HLH [34, 36, 37]	Disease relapse	
	Infections [24]	Infections [24]	
	Antibody‐mediated autoimmune cytopenias	Antibody‐mediated autoimmune cytopenias	
Possible factors associated	Grade 3–4 CRS/ICANS [17, 26, 27]	CAR construct* [26]	
	CAR construct [26]		
	Peak CRP/ferritin* [26]		
	Baseline cytopenia [32]		
	Number of prior lines of therapy [17, 26]		
	Prior allo‐BMT [25, 26]		
	Marrow tumor burden [17]		
Considerations for management	Evaluation for CAR‐HLH including ferritin, triglycerides, coagulopathy, bone marrow biopsy to asses for hemophagocytosis. Treatment with tocilizumab, anakinra+/−steroids [37]	Supportive care with transfusions, growth factors, TPO agonists [15, 16, 38, 46–48]	Marrow biopsy for primary disease or another malignancy such as MDS
	Evaluation for hemolysis, immune mediated thrombocytopenia	Disease evaluation for relapse	
	Evaluation and treatment of infections [24]	Marrow biopsy for disease or another malignancy such as MDS	

Abbreviations: BMT, bone marrow transplant; CAR, chimeric antigen receptor; CAR‐HLH, CAR T cell‐therapy‐related hemophagocytic lymphohistiocytosis; CRP, C‐ reactive protein; CRS, cytokine release syndrome; ICANS, immune effector cell‐associated neurotoxicity syndrome; MDS, myelodysplastic syndrome; TPO, thrombopoetin.

* Only studied in univariate analysis.

### 3.1 Early CAR‐T‐OPENIA (<30 days): *Quick as a flash*

As noted above, the cytopenias occur almost immediately following CAR T‐cell infusion, and median time to nadir often lies within a week. For the most part, these early CAR‐T‐OPENIA is attributed to lymphodepletion chemotherapy. The later remains an integral part of CAR T‐cell therapy as it enhances CAR T‐cell expansion, activation, and persistence possibly by increase in interleukin (IL)‐15 and depletion of regulatory T‐cells [30, 31, 32, 33]. However, lymphodepletion chemotherapy possibly exerts additional myelosuppressive effect on top of the bone marrow dysfunction from the underlying diagnosis, resulting in these early CAR‐T‐OPENIA [34]. Several studies, spanning various products and diagnoses, consistently suggest the correlation of higher‐grade CRS or ICANS with increased early CAR‐T‐OPENIA [18, 26, 27]. One possible reason for this is the higher interferon‐gamma levels in patients with higher‐grade CRS, which is known to negatively impact hematopoietic stem cell homeostasis resulting in exhaustion of this stem cell compartment [35]. However, a statistical difference in interferon‐gamma levels was not demonstrated in a limited analysis conducted to compare cytokine levels in patients with count recovery with those whose counts did not recovery by 1 month [26]. This discrepancy underscores the need for a systematic exploration of inflammatory cytokines and the pathobiology of this inflammatory milieu following CAR T‐cells in hematopoietic recovery. In addition to the above, infections including bacterial, fungal, or viral reactivations can play a perfect paradox, by being the cause or effect of early cytopenias [24]. Lastly, antibody‐mediated autoimmune cytopenias and thrombotic microangiopathy can also occur after CAR T‐cell therapy leading to at least transient CAR‐T‐OPENIA.

CAR‐associated hemophagocytic lymphohistiocytosis (CAR‐HLH) deserves a special mention, and consideration, in this early post‐CAR T‐cell phase. Like CRS, HLH is also an inflammatory syndrome which occurs from pathological T‐cell and macrophage activation. Hence, the CAR T‐cell CRS picture overlaps the commonly known clinical scenario of HLH including elevated ferritin levels, coagulopathy, liver dysfunction, and other end‐organ involvement (renal or pulmonary) [34, 36]. This CAR‐HLH can occur coinciding with CRS or following resolution of CRS [34]. A rising ferritin despite resolution of clinical symptoms of CRS can be indicative of ensuing CAR‐HLH following CRS [36]. Criteria for CAR‐HLH have been described by the pediatric group at National Cancer Institute as peak ferritin ≥100,000 μg/L with 2 of (a) hepatic transaminases or bilirubin ≥ 3, (b) creatinine ≥ grade 3 (c) pulmonary manifestation of edema or hypoxia, grade ≥ 3 (d) bone marrow evidence of hemophagocytosis (e) coagulopathy [37]. Specific evaluation for cytopenias where CAR‐HLH is a possible etiology should include the above, as also summarized in Table 2. Treatment can include CRS‐like management via IL‐6 inhibition with tocilizumab [34], while recent studies show elevation of interferon‐gamma and IL‐1beta following CRS, which prompts the role of anakinra with or without corticosteroids [37].

### 3.2 Short‐term CAR‐T‐OPENIA (30–90 days): *A test of patience*

As shown in the aforementioned studies, it is common for CAR‐T‐OPENIA to persist beyond 1 month. There are limited data to state factors that impact count recovery at 3 months. In the study from Memorial Sloan Kettering Cancer Center, only CAR construct was statistically associated with count recovery at 3 months, such that all patients who received tisagenlecleucel had count “recovery” at 3 months while only 42% patients who received axicabtagene ciloleucel did [26]. These results are best interpreted with caution due to a smaller sample size at 3 months (*n* = 41) in this study [26]. Treatable conditions mentioned in the “early” phase, such as infections, CAR‐HLH, or autoimmune cytopenias, can also occur in the “short‐term” and remain imperative to rule out or treat, if present. Disease persistence or relapse involving the marrow is a plausible reason for cytopenias in this early time‐frame and warrants evaluation.

Beyond the scope of the above addressable conditions, supportive care with the use of growth factors and/or thrombopoietin (TPO) agonists in addition to transfusions if needed is the mainstay. Granulocyte‐macrophage colony‐stimulating factor from CAR T‐cells has been implicated in the biology of CRS and ICANS [11, 16, 17], which resulted in at least a transient trepidation in using growth factors for the possibility of worsening CRS or ICANS. However, of late, increasing reports were published most of which demonstrate safety of using growth factors following CAR T‐cell therapy [38, 39], while one demonstrated an increase in severity of CRS [40]. Whether the later has meaningful clinical implications is unclear, and additional data will further clarify this enigma in future. In the meantime, a risk‐versus‐benefit balance discussion is warranted considering perils of prolonged neutropenia and risk of severe or recurrent infections.

The use of TPO agonists for CAR T‐cell‐related thrombocytopenia is anecdotal at this time [41]. Preclinical data support the role of TPO in proliferation and maintenance of hematopoietic stem cells [42, 43, 44]. Hence, TPO agonists have emerged as an attractive strategy for bone marrow failure syndromes and myelodysplastic syndromes [45, 46, 47]. While additional data will further elucidate the role of TPO agonists in CAR‐T‐OPENIA, the concept holds merit and remains worth exploring.

### 3.3 Prolonged CAR‐T‐OPENIA (>90 days): *And the saga goes on*

Much to our despair, CAR‐T‐OPENIA can persist beyond 3 months in some patients. While it is probably not outside the realms of imagination that lymphodepletion can result in prolonged CAR‐T‐OPENIA, marrow recovery would usually be anticipated by 3 months following lymphodepletion. Therefore, at this time marrow involvement with primary disease or a secondary marrow process is worth consideration. Myelodysplastic syndrome has been anecdotally reported following CAR T‐cell therapy [26, 48]. Whether this is related to CAR T‐cell therapy would be premature to state, especially in a setting where these patients have previously received chemotherapy that is well known to cause therapy‐related myeloid neoplasm. These include alkylating agents, platinum agents, and topoisomerase II inhibitors commonly used for treatment in lymphomas and ALL, or lenalidomide in multiple myeloma [49, 50]. A marrow biopsy is of utmost importance for evaluation of such marrow failure or dysplastic process contributing to these cytopenias.

## 4 ..AND AS WE MARCH FORWARD, FUTURE DIRECTIONS

As we have noted above, the exact mechanism of CAR‐T‐OPENIAs in these different time‐frames remains to be understood. This is imperative to be able to appropriately manage and prevent, if possible, such consequential CAR‐T‐OPENIA at bedside. This is also essential in instituting infection prophylaxis in these patients, which is currently guided by extrapolation from autologous or allogenic BMT at best. In this emerging era of earlier treatment and prevention of CAR T‐cell toxicities of CRS and ICANS, it will also be important to follow‐through with the rates of CAR‐T‐OPENIA when early treatment for CRS/ICANS is instituted. As depicted in this review, evidence‐based management options for each of these scenarios is rather limited, and more robust data, as would emerge with increasing use, will add to the important pool of evidence for use of therapies in CAR‐T‐OPENIA.

## CONFLICT OF INTEREST

Alankrita Taneja has no conflict of interest. Tania Jain reports institutional research support from CTI Biopharma and Syneos Health, Consultancy with Targeted Healthcare Communications, advisory board with Care Dx, and Bristol Myers Squibb.

## AUTHOR CONTRIBUTIONS

Alankrita Taneja and Tania Jain conceptualized the project, wrote, edited, revised, and finalized the manuscript.
